# Sevoflurane-Induced Thrombocytopenia in Post-Robotic-Assisted Paraesophageal Hernia Repair

**DOI:** 10.1155/2020/8851687

**Published:** 2020-09-21

**Authors:** Sara Alhaj, James Tran, Muhammad Nazim, Praveen Tumula, Hassan Ahmed

**Affiliations:** ^1^Texas Tech University Health Sciences Center School of Medicine, Amarillo, TX, USA; ^2^Department of Surgery, Texas Tech University Health Sciences Center School of Medicine, Amarillo, TX, USA; ^3^Texas Oncology, Amarillo, TX, USA

## Abstract

**Background:**

Transient transaminitis is an expected outcome from liver retraction after foregut surgeries. However, severe thrombocytopenia is usually not a sequela of that. We present a case in which sevoflurane is suspected of inducing thrombocytopenia as it was the only newly introduced medication to the patient during the hospital course. Thrombocytopenia may present in a variety of settings in hospitalized patients. However, managing this occurrence requires deep exploration of pathophysiology that can cause decreased platelets, which may be a challenging task in certain circumstances. The liver plays an important role in thrombopoiesis by releasing megakaryocyte growth factors. Therefore, liver dysfunction can present as thrombocytopenia or other platelet dysfunctions.

**Objective:**

To describe a presentation of thrombocytopenia possibly associated with anesthesia-induced transaminitis after a robotic paraesophageal hernia repair with mesh and fundoplasty with intraoperative esophagogastroduodenoscopy (EGD).

**Methods:**

A 55-year-old presented to the ED with abdominal pain and was found to have a large type IV paraesophageal hernia that was surgically treated with a robotic paraesophageal hernia repair with mesh. However, on the first postoperative day (POD) (#1), the patient developed new onset thrombocytopenia with transaminitis. Workup for thrombocytopenia failed to determine an etiology. With platelet transfusion, platelet count showed an upward trend. The patient was then evaluated and cleared for discharge by POD#5.

**Results:**

The patient's POD#1 daily labs showed elevated values for liver function tests and a low platelet count of 10,000 platelets per microliter with an international normalized value (INR) of 1.3. She had received two doses of intravenous acetaminophen just prior to surgery. Her platelet count responded to two units of platelets but decreased again immediately after. She continued to have transaminitis with down-trending liver enzymes. Peripheral smear on review showed no evidence of schistocytes. A heparin-induced thrombocytopenia (HIT) screening was negative. The patient was regularly evaluated, and the platelets stabilized and slowly started to trend up. The patient recovered by the morning of her POD#5 and was cleared for discharge.

**Conclusion:**

We are reporting on a case of acute postoperative thrombocytopenia that was associated with transaminitis and elevated liver enzymes. We are linking the role of the liver dysfunction in noncirrhotic patients with surgical abdominal procedures. Although liver retraction transaminitis possibly played a role in the laboratory findings in the patient, the acute drop in her platelet count could be closely related to the use of sevoflurane anesthetic considering its potential hepatotoxic side effects. We also cannot rule out the sevoflurane directly affecting the platelet count.

## 1. Introduction

Thrombocytopenia is defined as a platelet count less than 150,000 platelets/microliter which may be asymptomatic but can place the patient at risk of bleeding or can manifest as thrombotic events as well [1]. Thrombocytopenia is a commonly encountered condition in hospitalized patients regardless of the nature, duration, or severity of their illness. It can present as a challenge to treat due to the variety of the pathophysiology that can cause it. Platelets are produced in response to thrombopoietin (TPO) that is produced by the liver that can stimulate TPO receptors on hematopoietic stem cells to differentiate to platelet cells [1]. Therefore, the liver plays a vital role in the formation of platelets in the body. Many pathologies can affect the liver, which can be detected by elevated liver enzymes, hence impairing the functions that the liver secondarily controls. Upper gastrointestinal laparoscopic surgeries and the use of certain anesthetics have been linked to transient transaminitis with liver function impairment both of which are yet to be fully explained.

## 2. Case Presentation

**Figure 1:**
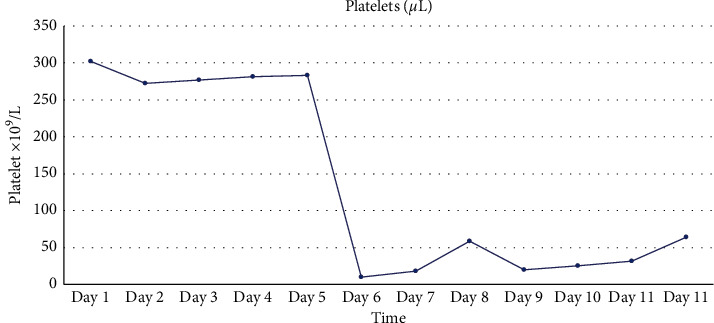
A graph depicting platelet count over time during this patient's hospital stay. The surgery was performed on hospital day 5.

**Figure 2:**
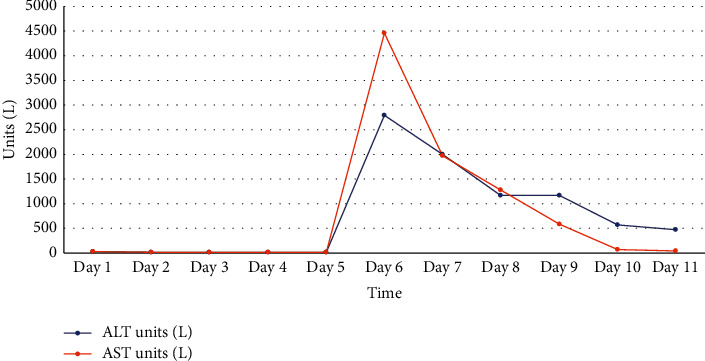
A graph depicting AST and ALT levels over time during this patient's hospital stay. Surgery was performed on hospital day 5.

A 55-year-old female with no known past medical history presented with abdominal pain and was found to have a large type IV paraesophageal hernia with approximately 75% of her stomach in the chest as well as the transverse colon with the presence of obstruction. She was found to be a candidate for a robotic paraesophageal hernia repair with mesh and fundoplasty and intraoperative EGD. The patient was taken to the operating room (OR), recovered well afterwards, and tolerated full liquids without any issues. However, on POD#1, the patient was found to have new onset thrombocytopenia which was not present prior to surgery. This trend is presented in Figure 1. The patient's platelets had dropped to 10,000 platelets/microliter with an INR of 1.3 (with normal range around 0.89–1.07), and complete metabolic panel was also significant for an aspartate transaminase (AST) level of 4461 and alanine transaminase (ALT) level of 2796, as shown in Figure 2; a normal AST level ranges around 8 to 48 units per liter (U/L), and a normal ALT level around 7 to 55 U/L. Her platelet count continued to trend down to as low as 9,000 platelets/microliter. She later received transfusion of 2 units of platelets which increased her platelets to 65,000 platelets/microliter which then abruptly decreased back to 18,000 shortly after. Peripheral smear showed no evidence of schistocytes, and a HIT screen was negative. The patient still showed transaminitis with down-trending liver enzymes and laboratory values significant for lactate dehydrogenase (LDH) of 2001 U/L (with normal range around 45–90 U/L), folate of 18 nmol/L (with normal range around 4.5–45.3 nmol/L), vitamin B12 of >1500 mg/L (with normal > 200 mg/L), fibrinogen of 220 mg/dL (with normal range around 200–400 mg/dL), INR of 1.5, prothrombin time (PT) of 17.9 s (with normal range around 11–15 s), and partial thromboplastin time (PTT) of 34 s (with normal range around 25–40 s). Bilirubin levels ranged between 0.3 and 1.3 mg/dL (with normal range around 0.1–1.2 mg/dL) throughout the hospitalization. Hemoglobin was 15.1 g/dL on admission with a gradual decrease to 11.0 g/dL (with normal range around 12.2–15.0 g/dL) during the hospitalization. No imaging was performed postoperatively to exclude causal relationship of a liver hematoma or other bleeding complication. Hepatitis C antibody was negative, and hepatitis B surface antibody (immune), surface antigens, and core antibody were negative. No bone marrow biopsy was performed. The patient did not have fever or C-reactive protein elevation, and no blood cultures were drawn. Ferritin was elevated at 402 *μ*g/mL on POD#2 (with normal range around 12–150 *μ*g/mL), but otherwise was within normal limits. Her platelets stabilized and slowly started to trend up with platelet transfusions, and the patient was evaluated the morning of her POD#5 and cleared for discharge.

## 3. Discussion

Reasons for thrombocytopenia can be grouped into the following broad categories: pseudothrombocytopenia, hemodilution, increased platelet consumption, increased platelet destruction, decreased platelet production, and increased platelet sequestration. However, we are presenting a perplexing case of postsurgical transaminitis and thrombocytopenia without a clear cause or predisposition. Prior to and during the patient's surgery, the patient exhibited liver enzyme values and platelets well within normal ranges. Paradoxically, this patient's symptoms of transaminitis and thrombocytopenia appear to have begun on POD#1 without a clear indication. There is literature that describes postsurgical platelet counts below 15,000 platelets/microliter following surgical procedures [2]. The nadir of the platelet count typically occurred within POD#1–4 with the platelets reaching normal ranges around POD#5[2]. This pattern seems to appear in major trauma, vascular, and abdominal surgeries as well as within populations of ICU patients [2]. It is also previously described that TPO introduction takes approximately three days to begin significantly increasing platelet counts [2].

From literature review, we hypothesize that this transient thrombocytopenia and transaminitis were a result of a brief slowdown of TPO production by the liver after sevoflurane exposure. The elevated liver enzymes seen on POD#1 may signify an acute irritation or damaging process of the liver. Acute irritation or damage to the liver may have resulted in hepatocellular slowing or halting due to an inflammatory or damaging process related to anesthetic use. As such, this would manifest mainly as an insufficient stimulation of megakaryocytes and thus inadequate platelet production [3]. Consequently, this would present as the profound thrombocytopenia as observed in this patient on POD#1 until the return to normal on POD#5.

This introduces the question as to what exactly factored into interrupted TPO production and why the liver was affected. On the one hand, hepatotoxicity is a risk of commonly used anesthetic agents in the United States. Sevoflurane, a commonly used anesthetic agent, has been implicated in cases of hepatotoxicity and at least one case of postoperative transaminitis with thrombocytopenia [4]. On the other hand, transient transaminitis has been associated with upper GI surgeries especially antireflux surgery [5]. This sequela has been linked to liver retraction during the procedure which was reported to resolve spontaneously with no clinical consequences. Although liver retraction plays a role in the postoperative transaminitis, the extent of the retraction during an upper GI surgery is irritation of segments II and III of the liver which constitute about 20–25% of its function [6]. The liver can meet its bodily function with up to 75% of its volume damaged [6]. Therefore, an extensive damaging process developed to cause the liver dysfunction which manifested in the form of significant transaminitis and consequent thrombocytopenia. After literature review and patient workup, sevoflurane anesthetic is the agent to cause the apparent fulminant hepatitis and liver dysfunction.

Finally, the discussion that sevoflurane caused these symptoms raises suspicion whether it was a direct effect on platelet function and aggregation or whether it was through hepatic toxicity that manifested in reduced TPO production and consequent thrombocytopenia. The patient's elevated liver enzyme resolved earlier than her platelet counts—therefore, drug interactions with platelets must be considered. Notably, halothane, sevoflurane, and propofol appear to reversibly inhibit platelet function in a dose-dependent manner, and sevoflurane's suppression of thromboxane A2 is suspected [7]. Propofol and nitrous oxide both suppress calcium mobilization which may contribute to an effect on decreasing platelet function [7]. However, some literature suggests that isoflurane, enflurane, desflurane, barbiturates, etomidate, opioids, and muscle relaxants do not appear to significantly affect platelet number or function [7]. In another study, volatile anesthetics such as sevoflurane caused increased platelet aggregation [8]. The study showed sevoflurane to increase antithrombotic effect and platelet aggregation within 15 minutes after intubation and anesthesia [8]. Isoflurane and desflurane showed reversibility after 15 minutes of induction, whereas sevoflurane showed continued effect past 1 hour after intubation. The study did not show, however, significant change in platelet count, PTT, or PT immediately after anesthesia induction [8]. On the contrary, sevoflurane has previously demonstrated hepatotoxicity and reproducible hepatic dysfunction after anesthesia in patients with no prior history of liver disease [9]. Sevoflurane is a relatively safe drug in its class; however, its induction of compound A production may play a role in liver insults [9]. Additionally, repeated exposure to halogenated volatile anesthetics such as sevoflurane may cause drug sensitization further manifesting as hepatotoxicity [9].

## 4. Conclusion

We conclude that anesthesia-induced hepatotoxicity occurs after minimally invasive upper GI surgeries and can have effects on processes controlled by the liver. We demonstrate the impairment of thrombopoiesis led by the liver which caused this thrombocytopenia. Other liver functions can also be affected by this transient transaminitis that can be symptomatic in other patients. Further studies are required for exploring the different implications of minimally invasive surgery and sevoflurane use on liver functions and the body as a whole. Although transaminitis is possibly due to liver retraction, literature indicates that it is commonly short-lived and recovers spontaneously [5]. In addition, the liver has the capacity to function with as low as 25% of its capacity [6]. Therefore, there is more evidence to indicate that sevoflurane anesthetic played a larger role in the fulminant liver failure and loss of functionality in inducing thrombopoiesis.
